# Plant Growth Promotion and Plant Disease Suppression Induced by *Bacillus amyloliquefaciens* Strain GD4a

**DOI:** 10.3390/plants13050672

**Published:** 2024-02-28

**Authors:** Piao Yang, Pu Yuan, Wenshan Liu, Zhenzhen Zhao, Matthew C. Bernier, Chunquan Zhang, Ashna Adhikari, Stephen Obol Opiyo, Lijing Zhao, Fredrekis Banks, Ye Xia

**Affiliations:** 1 Department of Plant Pathology, College of Food, Agricultural and Environmental Sciences, The Ohio State University, Columbus, OH 43210, USA; yang.4636@buckeyemail.osu.edu (P.Y.); liu.11241@osu.edu (W.L.); adhikari.168@buckeyemail.osu.edu (A.A.); opiyo.1@osu.edu (S.O.O.); ljzhao@ucdavis.edu (L.Z.); 2 Campus Chemical Instrument Center, Mass Spectrometry and Proteomics Facility, The Ohio State University, Columbus, OH 43210, USA; 3 College of Agriculture and Applied Sciences, Alcorn State University, Lorman, MS 39096, USA; czhang@alcorn.edu (C.Z.);

**Keywords:** *Arabidopsis thaliana*, bacterial extracellular exudates (BEE), benzocaine (BEN), *Botrytis cinerea*, metabolite analysis, systemic resistance

## Abstract

*Botrytis cinerea*, the causative agent of gray mold disease (GMD), invades plants to obtain nutrients and disseminates through airborne conidia in nature. *Bacillus amyloliquefaciens* strain GD4a, a beneficial bacterium isolated from switchgrass, shows great potential in managing GMD in plants. However, the precise mechanism by which GD4a confers benefits to plants remains elusive. In this study, an *A. thaliana*-*B. cinerea*-*B. amyloliquefaciens* multiple-scale interaction model was used to explore how beneficial bacteria play essential roles in plant growth promotion, plant pathogen suppression, and plant immunity boosting. *Arabidopsis* Col-0 wild-type plants served as the testing ground to assess GD4a’s efficacy. Additionally, bacterial enzyme activity and targeted metabolite tests were conducted to validate GD4a’s potential for enhancing plant growth and suppressing plant pathogens and diseases. GD4a was subjected to co-incubation with various bacterial, fungal, and oomycete pathogens to evaluate its antagonistic effectiveness in vitro. In vivo pathogen inoculation assays were also carried out to investigate GD4a’s role in regulating host plant immunity. Bacterial extracellular exudate (BEE) was extracted, purified, and subjected to untargeted metabolomics analysis. Benzocaine (BEN) from the untargeted metabolomics analysis was selected for further study of its function and related mechanisms in enhancing plant immunity through plant mutant analysis and qRT-PCR analysis. Finally, a comprehensive model was formulated to summarize the potential benefits of applying GD4a in agricultural systems. Our study demonstrates the efficacy of GD4a, isolated from switchgrass, in enhancing plant growth, suppressing plant pathogens and diseases, and bolstering host plant immunity. Importantly, GD4a produces a functional bacterial extracellular exudate (BEE) that significantly disrupts the pathogenicity of *B. cinerea* by inhibiting fungal conidium germination and hypha formation. Additionally, our study identifies benzocaine (BEN) as a novel small molecule that triggers basal defense, ISR, and SAR responses in Arabidopsis plants. *Bacillus amyloliquefaciens* strain GD4a can effectively promote plant growth, suppress plant disease, and boost plant immunity through functional BEE production and diverse gene expression.

## 1. Introduction

The Gram-positive bacteria *Bacillus amyloliquefaciens* have recently gained prominence as versatile microorganisms with applications spanning agriculture, medicine, and industry [1,2,3,4,5,6,7]. Various strains of *B. amyloliquefaciens* have been consistently isolated from diverse microbiomes, including the phyllosphere, endophytic environments, the rhizosphere, and soil microbiomes. These strains have been found with utility in agricultural and food chemistry by producing heterologous proteins, such as amylase, cellulase, keratinase, and protease [8,9,10,11,12,13], positioning *B. amyloliquefaciens* as a valuable resource for metabolic engineering in the synthesis of therapeutic proteins and related enzymes for industrial applications. Furthermore, certain secondary metabolites derived from *B. amyloliquefaciens* exhibit antagonistic activities against phytopathogens and possess traits beneficial for enhancing plant growth and bolstering host plant immunity against secondary pathogenic attacks [14,15,16,17,18,19,20]. However, the underlying mechanisms behind these effects remain unclear and can vary among different *B. amyloliquefaciens* strains. Consequently, *B. amyloliquefaciens* strains hold significant promises as candidates for the development of environmentally friendly and sustainable practices in agriculture, medicine, and industry [21,22,23,24].

Gray mold fungus, scientifically known as *Botrytis cinerea*, poses a significant threat to various plants, including vegetables, fruits, flowers, trees, and shrubs [25,26,27,28,29]. This fungal disease can strike at different stages of plant growth, targeting mature or senescent tissues, pre-harvest crops, or young seedlings [30,31,32]. Its widespread impact leads to substantial economic losses in both field and greenhouse cultivation [33,34,35,36,37]. Recent studies have unveiled the potential of *Bacillus* bacteria in managing gray mold disease. For instance, *Bacillus amyloliquefaciens* KRS005 has demonstrated the ability to inhibit the growth of *B. cinerea*, offering a promising alternative to chemical fungicides [38,39]. Bioactive secondary metabolites produced by *B. mojavensis* have promising applications in the agricultural, food, and clinical fields [40,41,42,43,44,45]. Additionally, *Bacillus proteolyticus* OSUB18 has been shown to activate the induced systemic resistance (ISR) against gray mold fungus in Arabidopsis while also directly impeding fungal growth [46,47]. On another front, *Pst* DC3000, a pathovar of *Pseudomonas syringae*, is recognized for causing bacterial speck disease in tomato and Arabidopsis plants [48,49,50,51]. It serves as a well-studied model pathogen for investigating disease susceptibility and hormone signaling in plants [52,53,54,55]. Although there exists comprehensive research on detecting, diagnosing, and managing the bacterial pathogen *Pseudomonas syringae* [53], there is a notable dearth of recent investigations into the specific use of *B. amyloliquefaciens* for controlling *Pst* DC3000. Consequently, further research is imperative to assess the efficacy of *B. amyloliquefaciens* against *Pst* DC3000 and to ascertain the optimal conditions for its deployment as a biocontrol agent in combatting this particular bacterial pathogen.

In a prior research endeavor, we successfully isolated *B. amyloliquefaciens* strain GD4a from the roots of switchgrass, extracted from a coal-mining site in Kentucky. Subsequent experiments confirmed its remarkable ability to enhance the growth of switchgrass plants [56]. Further investigation into its genome unveiled the presence of diverse gene clusters responsible for producing valuable metabolites, such as bacteriocin, fengycin, and indole 3-acetic acid [15], suggesting that GD4a holds promises not only in promoting plant growth but also in suppressing plant diseases and potentially modulating host plant immunity. However, the precise mechanisms through which GD4a enhances host plant fitness remain a subject of ongoing inquiry.

Traditional microbial and genomic methods have proven valuable for screening potential beneficial bacteria from the environments of host plants [15,57,58]. However, before these isolated beneficial bacteria can be effectively applied in agriculture, medicine, or industry, in-depth functional characterization is essential. In this study, we focus on the underexplored GD4a and comprehensively investigate its functional roles as a plant growth promoter, host immunity activator, and biological controller against a broad spectrum of plant pathogens, including bacteria, fungi, and oomycetes. Our primary objective is to validate GD4a’s beneficial traits in various host plant systems, including the model plant *Arabidopsis* and economically significant crops, such as tomatoes and strawberries. Specifically, we delve into how GD4a enhances *Arabidopsis’* ability to combat the devastating gray mold fungus, *Botrytis cinerea*. One of our central hypotheses is that GD4a can produce functional bacterial extracellular exudate (BEE) to activate plants’ ISR against this fungal pathogen’s infections. The other hypothesis is that BEE produced by GD4a can effectively disrupt the pathogenesis of the gray mold fungus by targeting its conidium development and hyphal formation. Our goal is to showcase the potential of GD4a and unravel the related functional mechanisms of GD4a in protecting crops not only during the growth season but also in post-harvest storage, particularly for fruits like tomatoes and strawberries. This includes safeguarding them from contamination by the gray mold fungus, both in the field and during transportation and storage processes. Furthermore, we hypothesize that, in addition to the direct pathogen inhibition, GD4a can reinforce the plant’s basal defense as well as trigger induced systemic resistance (ISR) through root-drenching treatments and potentially systemic acquired resistance (SAR) through leaf infiltration. ISR serves as a crucial systemic defense mechanism against pathogen infections in plants, generally activated upon interaction with specific beneficial microorganisms through root interactions. This form of defense confers broad-spectrum protection against a range of pathogens. The ISR mechanism predominantly operates through a signaling pathway where generally jasmonate and ethylene act as central mediators. This pathway orchestrates the activation of various defense responses throughout the plant, enhancing its overall resilience [59,60]. Unlike ISR through beneficial microbes, SAR is a sophisticated systemic plant defense mechanism that offers enduring protection against a wide array of pathogens through the activation of primary pathogen infections and chemical inducers. Activated by signaling molecules like salicylic acid (SA), SAR involves the systemic transmission of defense signals throughout the plants. Beyond salicylic acid, the mechanism is orchestrated by a complex network of different pathways, such as fatty acid, lipid, and amino acid pathways, highlighting its multifaceted nature [61,62,63]. Although our understanding of ISR and SAR in plants has advanced, there remain numerous mysteries surrounding the intricate pathways, mobile signals, and receptors involved. Ongoing research aims to unravel these complexities to enhance our knowledge of systemic plant defense mechanisms and their potential applications in agriculture. Essentially, GD4a, including its BEE, can be employed as a preemptive activator to prime host plants against potential future pathogen attacks. To address our research questions, we conducted experiments guided by data extracted from the GD4a genome. We assessed GD4a’s efficacy in promoting the fitness of *Arabidopsis* and conducted a series of comprehensive tests related to its specific metabolites analysis, enzyme activities, and potential plant genes targeted by GD4a. Additionally, GD4a BEE was employed to induce PAMP-Triggered Immunity (PTI) in *Arabidopsis*. We also extracted and purified GD4a BEE for untargeted metabolomic analysis, aiming to identify the functional antimicrobial compounds produced by GD4a. Moreover, we selected BEN, a compound identified in the untargeted metabolomic assay of GD4a BEE, for further investigation of its role in SAR in *Arabidopsis*.

Here, we detail our endeavors to elucidate the functions of GD4a in bolstering plant growth, quelling plant pathogens, and fortifying host plant immunity. We present substantiated findings that underscore GD4a’s role in fostering plant growth through the generation of advantageous metabolites and enzyme activities. Furthermore, GD4a demonstrates its capacity to activate the basal defense, ISR, and SAR mechanisms in host plants by orchestrating the regulation of plant defense gene expression. Additionally, GD4a exhibits proficiency in thwarting the pathogenicity of the gray mold fungus, primarily by impeding fungal conidium germination and hyphal formation. Importantly, GD4a’s efficacy extends to combatting a spectrum of fungal, oomycete, and bacterial phytopathogens by plate assays. These findings not only deepen our understanding of the intricate interactions between beneficial bacteria like GD4a and plants but also offer practical applications in agriculture for enhancing plant growth, fortifying plant defense mechanisms, and controlling devastating plant pathogens and diseases in both the growth season and post-harvest storage. These advancements hold promise for more sustainable and resilient agricultural practices in the future. This study’s future impact extends beyond agriculture to encompass biotechnological innovations, environmental conservation, and global efforts to address pressing challenges in food production and ecological sustainability. It paves the way for more resilient, eco-friendly, and sustainable agricultural practices and may hold the key to addressing some of the world’s most pressing agricultural and environmental issues.

## 2. Results

### 2.1. GD4a Enhances Plant Growth by Producing a Variety of Beneficial Metabolites

**Figure 1:**
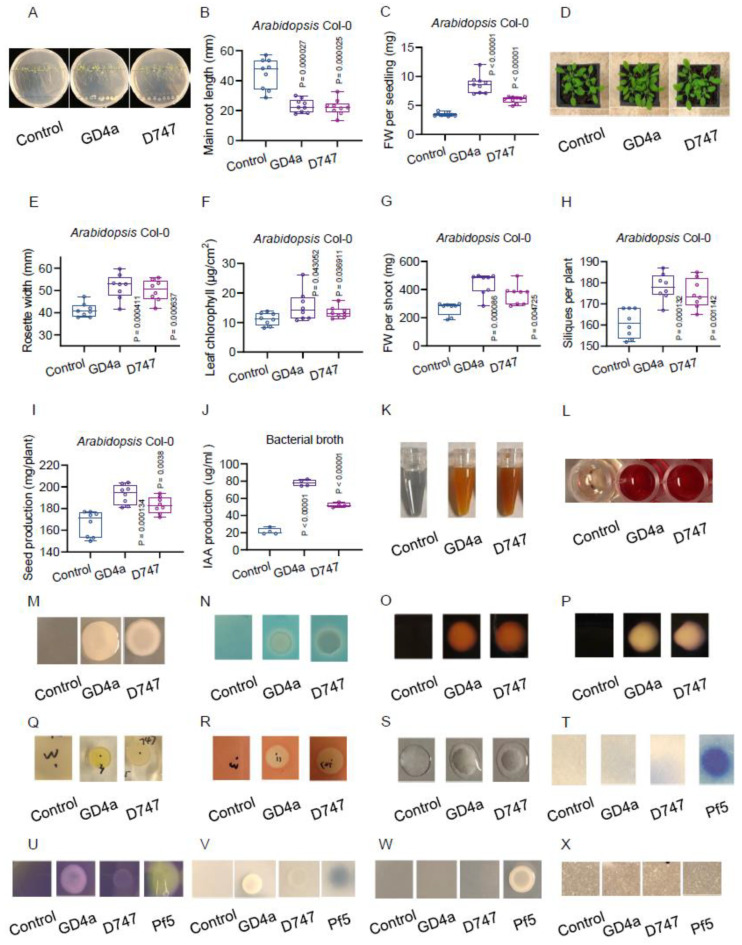
GD4a promotes plant growth by producing different metabolites and showing diverse enzyme activities. (**A**) GD4a inoculation increases the growth of *Arabidopsis* Col-0 seedlings on 1/2 MS media. (**B**) GD4a inoculation decreases primary root elongation of *Arabidopsis* Col-0 seedlings on 1/2 MS media. (**C**) GD4a inoculation increases the fresh-weight (FW) biomass of *Arabidopsis* Col-0 seedlings on 1/2 MS media. (**D**) GD4a drenching increases the growth of *Arabidopsis* Col-0 plants in soil. (**E**) GD4a drenching increases the rosette width of *Arabidopsis* Col-0 plants. (**F**) GD4a drenching increases the leaf chlorophyll content of *Arabidopsis* Col-0 plants. (**G**) GD4a drenching increases the shoot FW of *Arabidopsis* Col-0 plants. (**H**) GD4a drenching increases the silique production of *Arabidopsis* Col-0 plants. (**I**) GD4a drenching increases the seed production of *Arabidopsis* Col-0 plants. (**J**) GD4a produces IAA. (**K**) GD4a produces ammonia. (**L**) GD4a produces acetoin diacetyl. (**M**) GD4a produces exopolysaccharides. (**N**) GD4a produces siderophores. (**O**) GD4a is positive in pectinase activity. (**P**) GD4a is positive in amylase activity. (**Q**) GD4a is positive in ribonuclease activity. (**R**) GD4a is positive in cellulase activity. (**S**) GD4a catalases H_2_O_2_ to O_2_. (**T**) GD4a produces no detectable hydrogen cyanide. (**U**) GD4a produces no detectable organic acid. (**V**) GD4a is negative in phosphate solubilization activity. (**W**) GD4a is negative in ACC deaminase activity. (**X**) GD4a is negative in chitinase activity.

The Gram-positive bacterial endophyte *Bacillus amyloliquefaciens* strain GD4a was originally isolated from the roots of switchgrass plants collected from a coal-mining site in western Kentucky. This isolate exhibited favorable effects on the growth of switchgrass plants in a greenhouse study, leading to its selection for further study [15]. Notably, we discovered that GD4a’s ability to promote plant growth extended beyond switchgrass. For instance, GD4a inoculation significantly enhanced seedling growth in *Arabidopsis* Col-0 WT plants (Figure 1A), suggesting GD4a’s potential for enhancing crop growth through seed treatment. Upon GD4a treatment, we observed a decrease in the primary root length (Figure 1B), accompanied by a substantial increase in seedling fresh weight (Figure 1C). To further validate GD4a’s plant growth-promoting capabilities, we drenched soil-grown *Arabidopsis* Col-0 plants with GD4a cells and monitored the above-ground growth after three consecutive weeks of treatment post-transplanting (Figure 1D). As anticipated, GD4a-treated plants exhibited both larger (Figure 1E) and faster shoot growth (Figure 1F). The biomass of above-ground plant shoots increased by approximately 110% (Figure 1G). Consequently, *Arabidopsis* Col-0 plants treated with GD4a yielded approximately 20% more siliques (Figure 1H) and about 40% more seeds (Figure 1I) compared to the water control.

Considering that there was no direct physical contact between GD4a and *Arabidopsis* Col-0 plants (Figure 1A), it is highly plausible that GD4a’s influence on host plant growth was mediated through the production of soluble beneficial metabolites. Given the presence of genes in GD4a encoding various beneficial metabolites and enzymes [15], we posited that GD4a might produce metabolites/enzymes previously linked to plant growth promotion [46]. Our targeted metabolite tests unveiled GD4a’s production of IAA (Figure 1J), ammonia (Figure 1K), acetoin diacetyl (Figure 1L), exopolysaccharides (Figure 1M), and siderophores (Figure 1N), which are recognized as canonical beneficial metabolites and biofertilizer sources [46,64]. Furthermore, GD4a exhibited positive pectinase activity (Figure 1O), amylase activity (Figure 1P), ribonuclease activity (Figure 1Q), cellulase activity (Figure 1R), and catalase activity (Figure 1S), aligning with genomic data mining findings for GD4a [15]. However, GD4a did not show certain canonical beneficial traits, including hydrogen cyanide production (Figure 1T), organic acid production (Figure 1U), phosphate solubilization activity (Figure 1V), ACC deaminase activity (Figure 1W), and chitinase activity (Figure 1X). This implies the specific efficacy of GD4a in promoting plant growth.

### 2.2. GD4a Is an Effective Biological Control Agent against Gray Mold Pathogen B. cinerea

**Figure 2:**
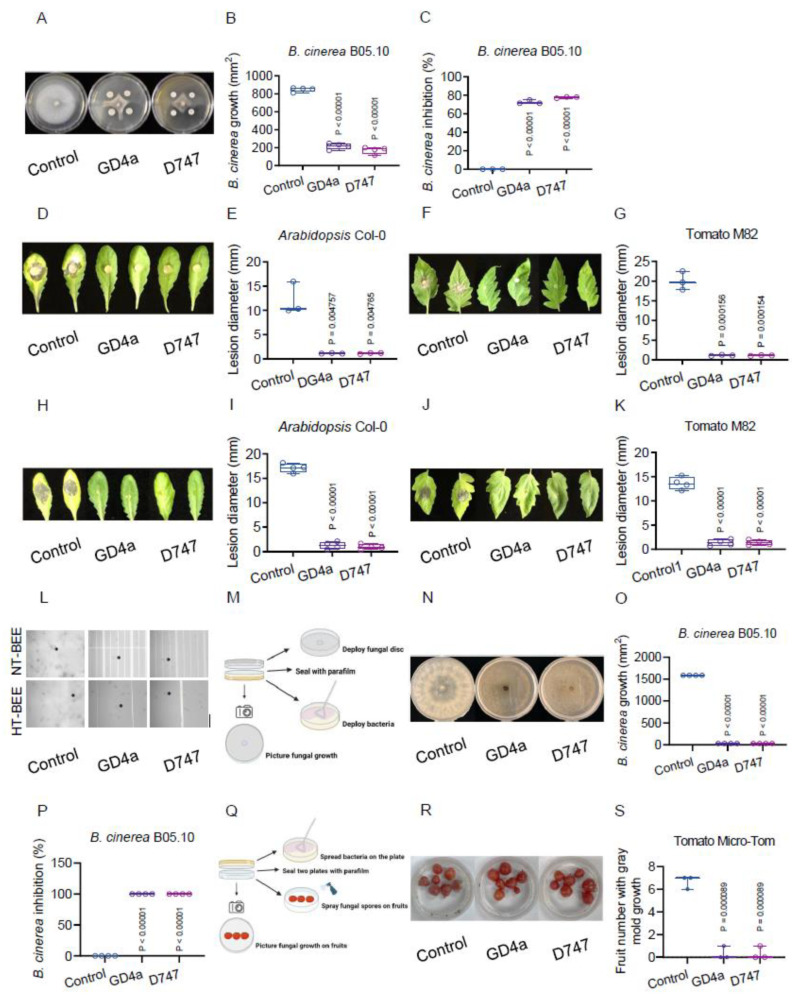
GD4a is an effective biological control against gray mold disease. (**A**) GD4a inhibits the growth of the gray mold fungus by the production of bacterial extracellular exudates (BEE). Please note that there is no direct contact between GD4a and *B. cinerea*, and the BEE is readily soluble in the agar media. (**B**) Quantification of the fungal growth of the assay in (**A**). (**C**) Quantification of the fungal inhibition rate of the assay in (**A**). (**D**) GD4a BEE leads to a pathogenicity loss of *B. cinerea* in *Arabidopsis* Col-0 plants. The mycelial discs for fungal infection were obtained from the assay in (**A**). (**E**) Quantification of the fungal disease symptom of the mycelial disc infection assay in (**D**). (**F**) GD4a BEE leads to a pathogenicity loss of *B. cinerea* in tomato M82 plants. The mycelial discs for fungal infection were obtained from the assay in (**A**). (**G**) Quantification of the fungal disease symptom of the mycelial disc infection assay in (**F**). (**H**) GD4a BEE leads to a pathogenicity loss of *B. cinerea* in *Arabidopsis* Col-0 plants. The fungal infection inoculum was a mix of fungal conidia with BEE of Control/GD4a/D747. (**I**) Quantification of the fungal disease symptom of the fungal conidium infection assay in (**H**). (**J**) GD4a BEE leads to a pathogenicity loss of *B. cinerea* in tomato M82 plants. The fungal infection inoculum was a mix of fungal conidia with BEE of Control/GD4a/D747. (**K**) Quantification of the fungal disease symptom of the fungal conidium infection assay in (**J**). (**L**) GD4a BEE inhibits the fungal conidium germination and hyphal formation of *B. cinerea*. HT-BEE, heat-treated BEE (65 °C for 20 min). NT-BEE, nontreated BEE. Representative conidia were pinpointed by black star icons. (**M**) Illustration of the assay to evaluate GD4a’s biological control against *B. cinerea* due to the production of volatile bacterial extracellular exudates (VBEE). Created with BioRender.com. (**N**) GD4a VBEE (VOCs) block the fungal growth of *B. cinerea*. (**O**) Quantification of the fungal growth of the assay in (**N**). (**P**) Quantification of the fungal inhibition rate of the assay in (**N**). (**Q**) Illustration of the assay to evaluate GD4a protecting post-harvested tomato fruits against *B. cinerea* by VBEE. Created with BioRender.com. (**R**) GD4a VBEE protects post-harvested tomato fruits from the gray mold. (**S**) Quantification of the gray mold incidence of the fungal conidium infection assay in (**R**).

To delve deeper into GD4a’s potential for plant protection, we conducted an antagonistic assay to assess its effectiveness against *B. cinerea*, a notorious fungal pathogen causing gray mold disease. Strikingly, GD4a exhibited a significant inhibition of *B. cinerea* growth in vitro (Figure 2A,B), resulting in an impressive inhibition rate of approximately 70% (Figure 2C). Notably, when co-incubated with GD4a, *B. cinerea* fungal discs (Figure 2A) lost much of their pathogenicity both on the research model plant *Arabidopsis* (Figure 2D,E) and the economically significant crop plant tomato (Figure 2F,G) in vivo. This highlights GD4a’s great potential as a promising biological control agent against the devastating gray mold disease.

To further validate GD4a’s efficacy against the gray mold fungus, we conducted an additional layer of the antagonistic assay. In this approach, GD4a cells were mixed with the fungal conidium inoculum for the fungal infection assay in plants. Notably, both *Arabidopsis* plants and tomato plants displayed minimal disease symptoms after *B. cinerea* infection, in contrast to the control groups (Figure 2H–K). Given the absence of a direct physical interaction between GD4a and *B. cinerea* in the previous in vitro co-plate assay (Figure 2A), we inferred that GD4a’s bacterial extracellular exudate (BEE) played a pivotal role in antagonizing the fungal pathogen. Indeed, GD4a’s BEE effectively inhibited fungal conidia germination observed under a microscope, thereby impeding infectious hyphae formation. Importantly, GD4a’s BEE also demonstrated heat stability (Figure 2L), indicating its promising potential for field applications relating to global warming.

Surprisingly, the volatile bacterial extracellular exudates (VBEE) released by GD4a also exhibited robust inhibitory effects on *B. cinerea* growth (Figure 2M–O), achieving an impressive inhibition rate of approximately 100% (Figure 2P). This intriguing discovery introduces a novel strategy for utilizing beneficial bacteria in gray mold disease control, particularly in scenarios like post-harvest fruit conservation and transportation. To validate this innovative concept, we designed an assay to assess GD4a’s ability to protect harvested tomato fruits against *B. cinerea* using VBEE (Figure 2Q). As anticipated, GD4a’s VBEE effectively curbed the proliferation of *B. cinerea* conidium contamination on post-harvested tomato fruits (Figure 2R,S).

### 2.3. GD4a Is an Effective Biological Control against Diverse Fungal, Oomycete, and Bacterial Phytopathogens

**Figure 3:**
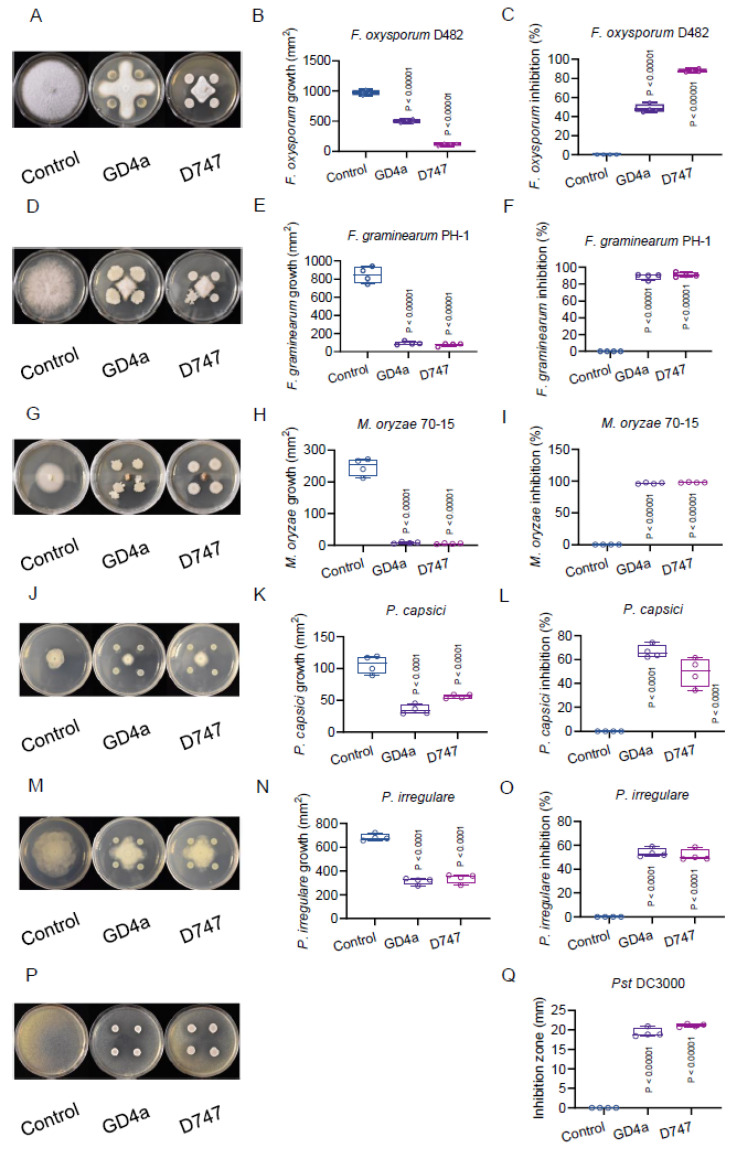
GD4a is an effective biological control against vascular wilt disease, fusarium head blight disease, rice blast disease, fruit rot disease, damping off disease, and bacterial speck disease. (**A**) GD4a inhibits the growth of *F. oxysporum* in vitro. (**B**) Quantification of the growth of *F. oxysporum* in (**A**). (**C**) Quantification of the inhibition rate of *F. oxysporum* in (**A**). (**D**) GD4a inhibits the growth of *F. graminearum* in vitro. (**E**) Quantification of the growth of *F. graminearum* in (**D**). (**F**) Quantification of the inhibition rate of *F. graminearum* in (**D**). (**G**) GD4a inhibits the growth of *M. oryzae* in vitro. (**H**) Quantification of the growth of *M. oryzae* in (**G**). (**I**) Quantification of the inhibition rate of *M. oryzae* in (**G**). (**J**) GD4a inhibits the growth of *P. capsici* in vitro. (**K**) Quantification of the growth of *P. capsici* in (**J**). (**L**) Quantification of the inhibition rate of *P. capsici* in (**J**). (**M**) GD4a inhibits the growth of *P. irregulare* in vitro. (**N**) Quantification of the growth of *P. irregulare* in (**M**). (**O**) Quantification of the inhibition rate of *P. irregulare* in (**M**). (**P**) GD4a inhibits the growth of *Pst* DC3000 in vitro. (**Q**) Quantification of the inhibition zone of *Pst* DC3000 in (**P**). Please note that there is no direct contact between GD4a and the corresponding plant pathogen. The GD4a BEE is readily soluble in the agar media.

Significantly, GD4a has demonstrated promising potential in controlling a wide range of phytopathogens, including fungi, oomycetes, and bacteria. In the antagonistic assay between GD4a and *F. oxysporum*, the causal agent of plant vascular wilt disease [34,65], GD4a achieved an inhibition rate of approximately 50% against the fungal pathogen in vitro (Figure 3A–C). Similarly, when tested against *F. graminearum*, responsible for wheat head blight disease [34,66], GD4a exhibited an impressive inhibition rate of around 90% in vitro (Figure 3D–F). Against *M. oryzae*, the causal agent of rice blast disease [34,67], GD4a displayed a remarkable inhibition rate of about 95% in vitro (Figure 3G–I). Turning to oomycete pathogens, the antagonistic assay between GD4a and *P. capsici*, which causes plant fruit rot disease [68], yielded an inhibition rate of approximately 70% in vitro (Figure 3J–L). Similarly, when confronted with *P. irregulare*, responsible for plant damping off disease [68], GD4a exhibited an inhibition rate of roughly 50% against the oomycete pathogen in vitro (Figure 3M–O). Lastly, GD4a’s effectiveness extended to bacterial pathogens, as shown in the antagonistic assay with *P. syringae*, which causes plant bacterial speck disease [53,69]. In this case, GD4a produced an inhibition zone of approximately 20 mm against the bacterial pathogen in vitro (Figure 3P,Q).

### 2.4. Induction of ISR and SAR of Host Plants against Fungal and Bacterial Pathogens by GD4a through BEE Production

**Figure 4:**
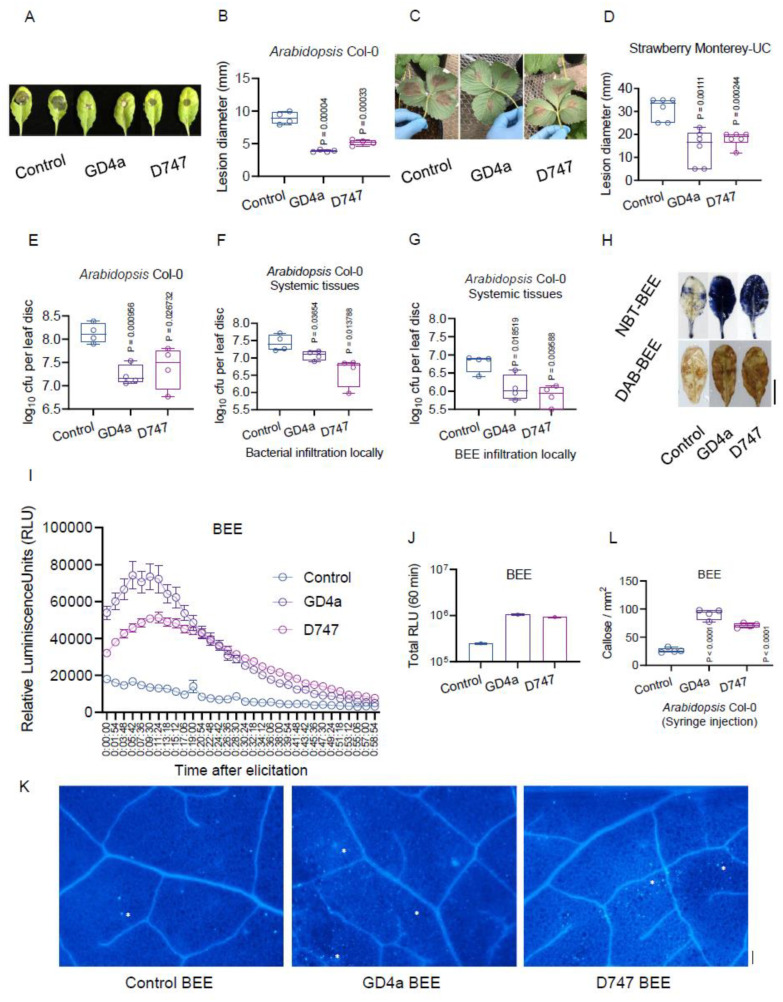
GD4a activates ISR against fungal and bacterial pathogens, and GD4a BEE activates SAR against fungal and bacterial pathogens. (**A**) GD4a root drenching protects *Arabidopsis* Col-0 plants against the gray mold fungus *B. cinerea*. (**B**) Quantification of the fungal disease symptom of the assay in (**A**). (**C**) GD4a root drenching protects strawberry Monterey plants against the gray mold fungus *B. cinerea*. (**D**) Quantification of the fungal disease symptom of the assay in (**C**). (**E**) GD4a root drenching protects *Arabidopsis* Col-0 plants against the bacterial pathogen *Pst* DC3000. (**F**) GD4a local leaf infiltration activates SAR against the bacterial pathogen *Pst* DC3000. (**G**) Local leaf infiltration of GD4a BEE activates SAR against the bacterial pathogen *Pst* DC3000. (**H**) GD4a BEE infiltration induces ROS production in *Arabidopsis* Col-0 plants. (**I**) GD4a BEE induces ROS burst of leaf discs in *Arabidopsis* Col-0 plants. (**J**) Quantification of the ROS burst of the assay in (**I**). (**K**) GD4a BEE infiltration induces callose deposition in *Arabidopsis* Col-0 leaves. Representative callose was pinpointed by white star icons. (**L**) Quantification of the callose deposition in (**K**).

In addition to their direct impact on inhibiting pathogens, beneficial bacteria also indirectly manage plant pathogens by modulating host plant immunity through mechanisms like ISR and SAR [46,47,57,58]. ISR and SAR are two different types of systemic plant defense mechanisms. Generally, ISR is induced by microorganisms (such as bacteria and fungi, which colonize the plant’s roots or leaves). SAR is activated by the pathogens and chemicals (such as bacteria and fungi, which infect the plant’s leaves; or metabolites/chemicals). Both ISR and SAR provide broad-spectrum protection against pathogens and involve the activation of various defense pathways in the plants. To delve deeper into GD4a’s efficacy in controlling gray mold disease, we applied GD4a through a drenching treatment (conducted weekly for a total of three times) on soil-grown *Arabidopsis* Col-0 and strawberry Monterey-UC plants. These were compared against water (negative control), GD4a, or D747 (positive control, *Bacilus amyloliquefaciens* strain D747), followed by the inoculation of above-ground leaf tissues with *B. cinerea* fungal conidia. The outcomes showed that GD4a significantly induced ISR responses in both *Arabidopsis* (Figure 4A,B) and strawberry (Figure 4C,D) plants against the gray mold fungus. Our recent findings also highlighted that the beneficial bacterium OSUB18 effectively triggered ISR against various fungal and bacterial pathogens in *Arabidopsis* through root drench treatment [46]. This prompted our hypothesis that GD4a can also activate host plant immunity against a diverse range of plant pathogens. Indeed, GD4a drenching had a notable impact, significantly bolstering *Arabidopsis* immunity against the bacterial pathogen *Pst*DC3000 (Figure 4E).

In a surprising revelation, both GD4a itself and its BEE (bacterial extracellular exudate) initiated the effective SAR in *Arabidopsis* Col-0 plants against *Pst*DC3000 through the leaf infiltration assay (Figure 4F,G), indicating the remarkable potential of GD4a’s BEE. Further investigation into GD4a’s BEE’s role in activating host plant immunity focused on the canonical plant PTI responses, including ROS production, ROS burst, and callose deposition. *Arabidopsis* Col-0 leaves infiltrated with the BEE of GD4a exhibited higher ROS production (Figure 4H), more rapid and robust ROS bursts (Figure 4I,J), and increased callose deposition (Figure 4K,L) compared to control plants. These findings shed light on the mechanisms behind the SAR triggered by GD4a (Figure 4F) and GD4a’s BEE (Figure 4G) in wild-type *Arabidopsis* Col-0 plants.

### 2.5. BEE of GD4a Has the Direct Pathogen Inhibition Activities by the Plate Assay

**Figure 5:**
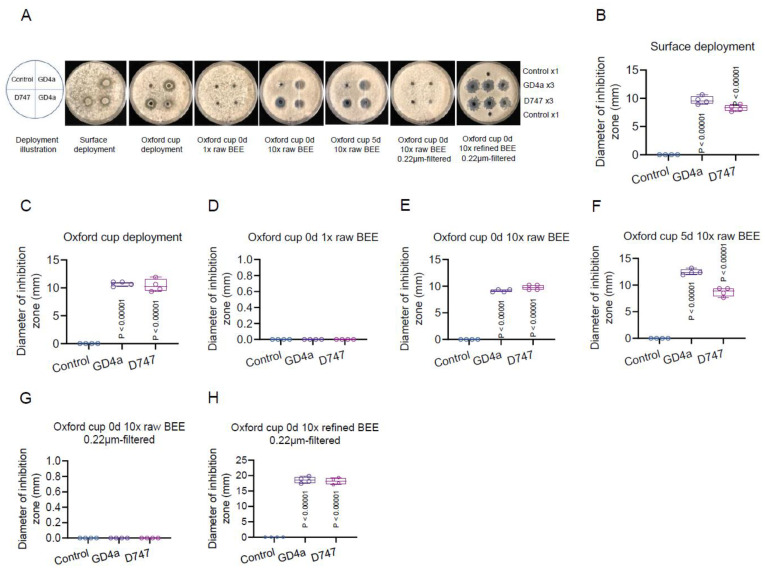
Untargeted metabolomics analysis of GD4a BEE. (**A**) GD4a BEE inhibits the gray mold fungus growth in vitro. (**B**) Quantification of the fungal growth inhibition of the assay in (**A**) as of surface deployment of alive GD4a cells. (**C**) Quantification of the fungal growth inhibition of the assay in (**A**) as of the Oxford cup deployment of alive GD4a cells. (**D**) Quantification of the fungal growth inhibition of the assay in (**A**) as of the Oxford cup deployment of fresh 1× raw BEE of GD4a. (**E**) Quantification of the fungal growth inhibition of the assay in (**A**) as of the Oxford cup deployment of fresh 10× raw BEE of GD4a. (**F**) Quantification of the fungal growth inhibition of the assay in (**A**) as of the Oxford cup deployment of 5-day-old 10× raw BEE of GD4a. (**G**) Quantification of the fungal growth inhibition of the assay in (**A**) as of the Oxford cup deployment of fresh 10× raw BEE of GD4a after running through a 0.22 µm filter. (**H**) Quantification of the fungal growth inhibition of the assay in (**A**) as of the Oxford cup deployment of fresh 10× refined BEE of GD4a after running through a 0.22 µm filter.

To delve deeper into GD4a’s effectiveness in countering *B. cinerea*, we initiated an inoculation study by evenly distributing fresh fungal conidia on agar plates. Subsequently, we introduced GD4a or its BEE onto the plate (Figure 5A, 1st panel). After a two-day incubation at room temperature, we observed a distinct inhibition zone where GD4a countered *B. cinerea* growth on the plate (Figure 5A, 2nd panel, Figure 5B). Employing the Oxford cup technique resulted in an even clearer inhibition zone while ensuring no direct contact between GD4a cells and the fungal conidia (Figure 5A, 3rd panel, Figure 5C), underscoring the significance of BEE. However, the original concentration of raw BEE did not display a noticeable inhibition zone (Figure 5A, 4th panel, Figure 5D), possibly due to the relatively low concentration of functional metabolites within the BEE. Notably, an evident inhibition zone emerged upon escalating the BEE concentration from 1× to 10× (Figure 5A, 5th panel, Figure 5E). Intriguingly, both the 0-day and 5-day 10× raw BEE (Figure 5A, 6th panel, Figure 5F) exhibited similar efficacy in antagonizing fungal growth, indicating a slow-decay characteristic of the BEE. This feature suggests its potential applicability in real-world conditions. However, due to the high concentration of the 10× BEE causing clogging issues in the 0.22 μm filter, we were unable to assess the effectiveness of the filter’s output in antagonizing *B. cinerea* (Figure 5A, 7th panel, Figure 5G). Fortunately, this filtration issue was resolved by incorporating a methanol purification step prior to filtering. The 10× refined BEE demonstrated a clear inhibition zone (Figure 5A, 8th panel, Figure 5H), affirming the presence of functional metabolites in the purified BEE.

To elucidate the functional metabolites produced by GD4a and D747, we conducted an untargeted metabolomics assay on their 10× refined BEE. In both negative and positive ion modes (normalized compound abundance > 1,000,000), we found that the majority of compounds comprised amino acids, alkaloids, flavonoids, and saponins (unpublished data). Considering those amino acids, alkaloids, flavonoids, and saponins are distinct classes of plant-derived secondary metabolites with diverse roles in enhancing plant defense against pathogens and other environmental stressors, it is intriguing to observe the prolific production of these compounds by beneficial bacteria. The abundant synthesis of amino acids, alkaloids, flavonoids, and saponins in GD4a’s BEE may substantiate its remarkable potential in promoting plant growth and curbing plant pathogens and diseases, which needs further investigation in the future.

### 2.6. Benzocaine (BEN) Induces Host Plant Systemic Resistance to Bacterial and Fungal Pathogens

**Figure 6:**
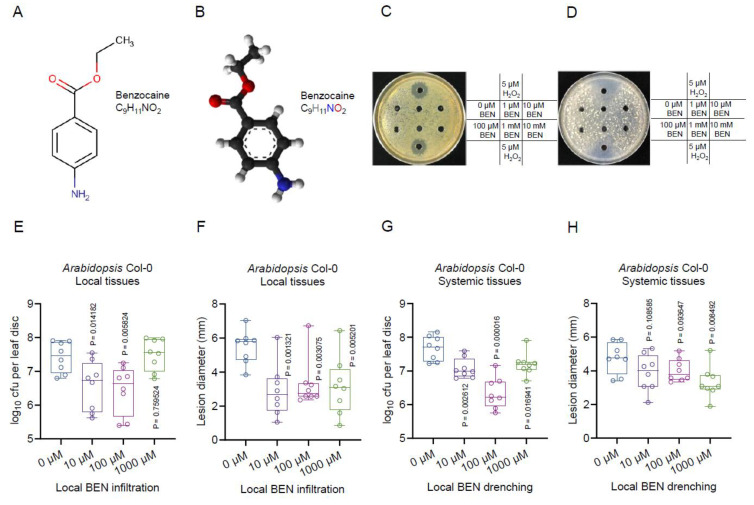
BEN (C9H11NO2) confers resistance to bacterial and fungal pathogens. (**A**) The 2D structure of BEN. (**B**) The 3D structure of BEN. (**C**) BEN shows no antimicrobial activity to bacterial pathogen *Pst*DC3000. (**D**) BEN shows no antimicrobial activity to fungal pathogen *B. cinerea* B05.10. (**E**) BEN-induced resistance against *Pst*DC3000 is concentration-dependent. (**F**) BEN-induced resistance against *B. cinerea* B05.10 at different concentrations. (**G**) BEN drenching-induced resistance against *Pst*DC3000. (**H**) BEN drenching-induced resistance against *B. cinerea* B05.10.

Importantly, our analysis revealed the identification of a compound, 4.01_165.0779n, detected in both the negative and positive ion modes with the formula C_9_H_11_NO_2_. There are 10 distinct isomers or HMDB IDs sharing the same formula C_9_H_11_NO_2_, including an intriguing chemical entity known as benzocaine (BEN), which was chosen for our further study. Notably, BEN’s role in activating host plant systemic immunity against phytopathogens remains unexplored. Benzocaine, recognized as a local anesthetic belonging to the amino ester drug class, finds utility in providing temporary relief from pain and itching associated with minor burns, sunburn, scrapes, insect bites, or minor skin irritations (Figure 6A). Its application spans topical pain alleviation and cough drops, often serving as the active ingredient in over-the-counter anesthetic ointments, such as those used for oral ulcers (Figure 6B). BEN does not demonstrate direct pathogen-killing capabilities against bacterial (Figure 6C) and fungal (Figure 6D) pathogens in the in vitro plate assays. Instead, its potential contribution lies in modifying plant immune responses. Our hypothesis centers around BEN’s ability to influence plant reactions to pathogen infections and other stresses. We aimed to ascertain whether BEN can fortify plants against bacterial and fungal pathogens. By injecting varying concentrations of BEN to *Arabidopsis* Col-0 leaves five hours before introducing them to the *Pst*DC3000 bacteria or *B. cinerea* B05.10 fungus, we observed the heightened plant resistance to both pathogens in vivo (Figure 6E,F). Furthermore, applying BEN to the roots as the trench treatment resulted in the enhanced defense of the above-ground sections against the pathogens (Figure 6G,H), illustrating BEN’s versatile role in enhancing plant immunity. To our knowledge, this is the first documented instance illustrating the efficacy of BEN in bolstering plant immunity.

### 2.7. Exogenous BEN-Infiltration Induces Local Defense Responses in Arabidopsis

**Figure 7:**
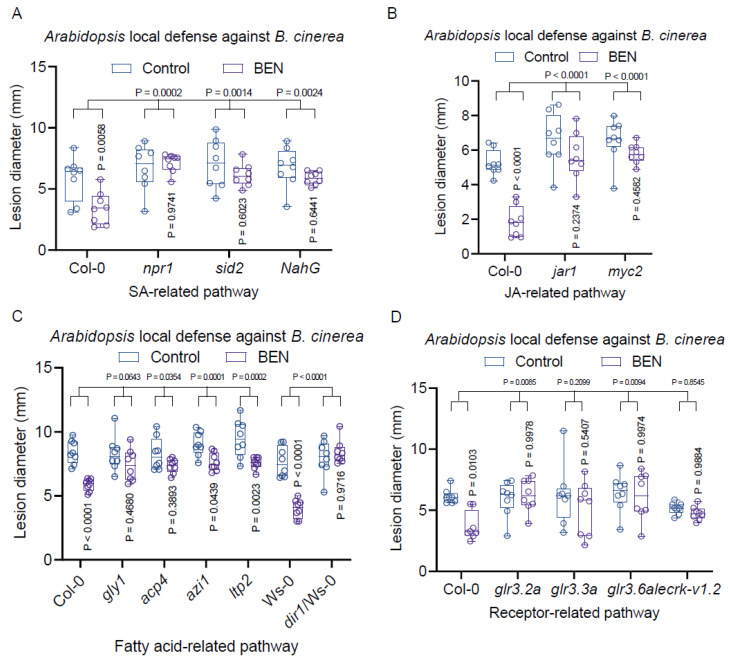
Exogenous BEN-induced local defense response in *Arabidopsis*. (**A**) The SA signaling pathway is required for BEN-mediated local plant immunity. (**B**) The JA signaling pathway is necessary for BEN-facilitated local plant immunity. (**C**) *ACP4* and *DIR1*, which are involved in the fatty acid and lipid signaling pathway, are vital for BEN-interceded local plant immunity. (**D**) *GLR3.2A* and *GLR3.6A* may be involved in BEN-mediated local plant immunity.

We then delved into the mechanisms underlying BEN’s activation of local plant defense in *Arabidopsis*. In our pursuit of understanding the role of salicylic acid (SA) and jasmonic acid (JA) signaling pathways in BEN-induced local plant defense, we conducted the following experiments. Infiltrating leaves of *Arabidopsis* wild type and mutants with defective SA or JA signaling pathways with 100 µM of BEN, we subjected them to a challenge with the *B. cinerea* B05.10 fungus five hours later. Subsequently, we assessed disease symptoms and fungal growth in the infected leaves 2–3 days afterward. These results were compared to those obtained from wild-type Col-0 plants treated with BEN. The outcome indicated that BEN’s ability to enhance plant resistance was less pronounced in both SA and JA mutants compared to the response in wild-type plants (Figure 7A,B). This underscores the essential involvement of both SA and JA signaling pathways in BEN-mediated local plant immunity.

Further probing the intricate network of *Arabidopsis* defense responses against the fungal pathogen *B. cinerea*, we explored the engagement of fatty acids and lipids as well as the chemical receptor signaling pathways facilitated by BEN. Fatty acids and lipids can serve critical roles in cellular signaling pathways, where they modulate enzyme activities and act as secondary messengers. By binding to a diverse array of receptors, fatty acids and lipids can initiate a multitude of downstream signaling events. The complexity of fatty acid and lipid signaling is underscored by the intricate interplay between various proteins and receptors and contributes to SAR [70,71,72]. GLR3.2a, GLR3.3a, and GLR3.6a serve as key members of the Glutamate Receptor-Like (GLR) gene family in Arabidopsis thaliana. These genes play multifaceted roles in the plant’s responses to both biotic and abiotic stresses, including insect herbivory, physical wounding, and carbon/nitrogen sensing. Notably, GLR3.2a, GLR3.3a, and GLR3.6a have been shown to mediate systemic resistance against insect herbivores in Arabidopsis, primarily by modulating the jasmonic acid and glucosinolate signaling pathways [73]. Employing the same inoculation procedure outlined above, we assessed the disease resistance of wild-type plants and mutants with defects in these pathways (namely *gly1*/*acp4/azi1/ltp2/dir1* for fatty acid and lipid pathways; and *glr3.2a*, *glr3.3a*, *glr3.6a*, and *lecrk-v1.2* for the GLR family). Our findings demonstrated that BEN substantially activated the resistance of wild-type plants, but its effect was notably subdued in most of the related mutants, such as *acp4*, *dir1*, *glr3.2a*, and *glr3.6a* (Figure 7C,D). This underscores the potentially critical role of fatty acids and lipids as well as chemical receptor signaling pathways in the BEN-aided local plant defense within *Arabidopsis*.

### 2.8. Exogenous BEN-Infiltration Induces Systemic Defense Response in Arabidopsis

**Figure 8:**
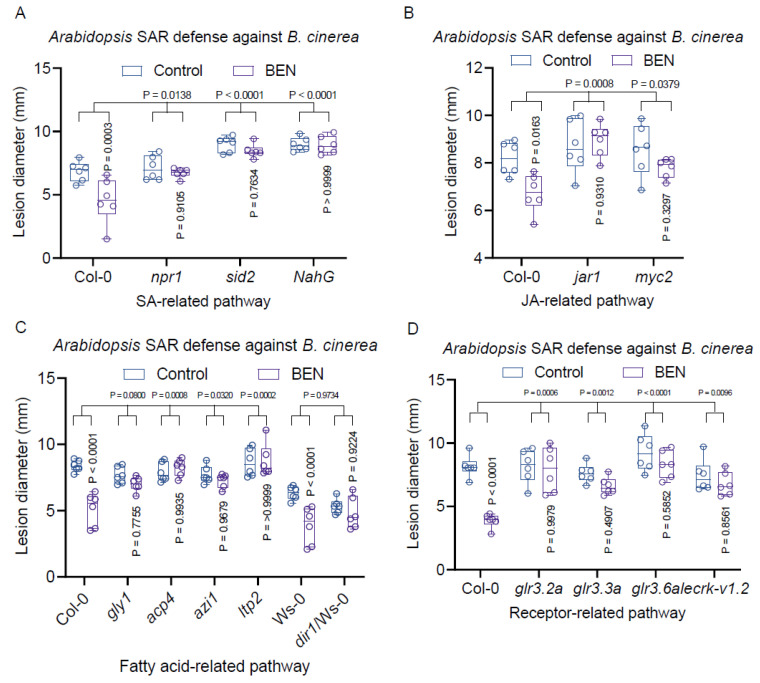
The exogenous BEN-induced systemic defense response in *Arabidopsis*. (**A**) The SA signaling pathway is required for BEN-mediated systemic plant immunity. (**B**) The JA signaling pathway is necessary for BEN-facilitated systemic plant immunity. (**C**) The *ACP4*, *AZI1*, and *LTP2* genes involved in fatty acid and lipid signaling are vital for BEN-interceded systemic plant immunity. (**D**) The chemical receptor pathway may be involved in BEN-mediated systemic plant immunity.

Our exploration extended to understanding the mechanisms through which BEN activates SAR in *Arabidopsis*. Initially, we scrutinized the involvement of SA and JA signaling pathways in the BEN-triggered systemic plant defense in *Arabidopsis*. To this end, we infiltrated 1 mM of BEN into the local leaves of *Arabidopsis* mutants afflicted with impairments in either SA or JA signaling pathways. After 48 h, we introduced untreated systemic leaves to the necrotrophic fungus *B. cinerea* B05.10. Subsequently, we assessed disease symptoms and fungal growth in the systemically infected leaves, comparing them with those in wild-type plants subjected to local BEN treatment. Our findings revealed that BEN’s capacity to enhance systemic plant resistance was notably diminished in SA or JA mutants compared to the response in wild-type plants (Figure 8A,B). This underscores the indispensable role of both SA and JA signaling pathways in BEN-mediated systemic plant immunity.

Subsequently, we delved into the role of fatty acids and lipids as well as chemical receptor signaling pathways in *Arabidopsis*’ systemic defense responses engendered by BEN against *B. cinerea*. LecRK-VI.2 is a prominent member of the Lectin Receptor-Like Kinase (LecRLK) gene family in Arabidopsis thaliana, integral to the plant’s defense mechanisms against a range of pathogens. Recent research from the University of Florida has illuminated LecRK-VI.2’s role as a potential receptor for extracellular NAD+ (eNAD+) and NAD+ phosphate (eNADP+). The study suggests that LecRK-VI.2 is central to the biological induction of systemic acquired resistance (SAR), expanding our understanding of plant defense pathways [74]. Employing the same inoculation methodology outlined earlier, we gauged the disease resistance of wild-type plants and mutants with deficiencies in these pathways (namely, *gly1*, *acp4*, *azi1*, *ltp2*, *dir* and *glr3.2a*, *glr3.3*, *glr3.6a*, *lecrk-v1.2*). Our observations demonstrated that BEN markedly augmented the systemic resistance of wild-type plants, yet its impact was notably subdued in most of these mutants, including *acp4*, *azi1*, *ltp2*, *glr3.2a*, *glr3.3*, *glr3.6a*, and *lecrk-v1.2* (Figure 8C,D). This highlights the pivotal role played by fatty acids and lipids as well as chemical receptor signaling pathways in BEN-facilitated systemic plant defense in *Arabidopsis*.

### 2.9. Exogenous BEN-Infiltration Induced Plant Defense Gene Expression in Arabidopsis

**Figure 9:**
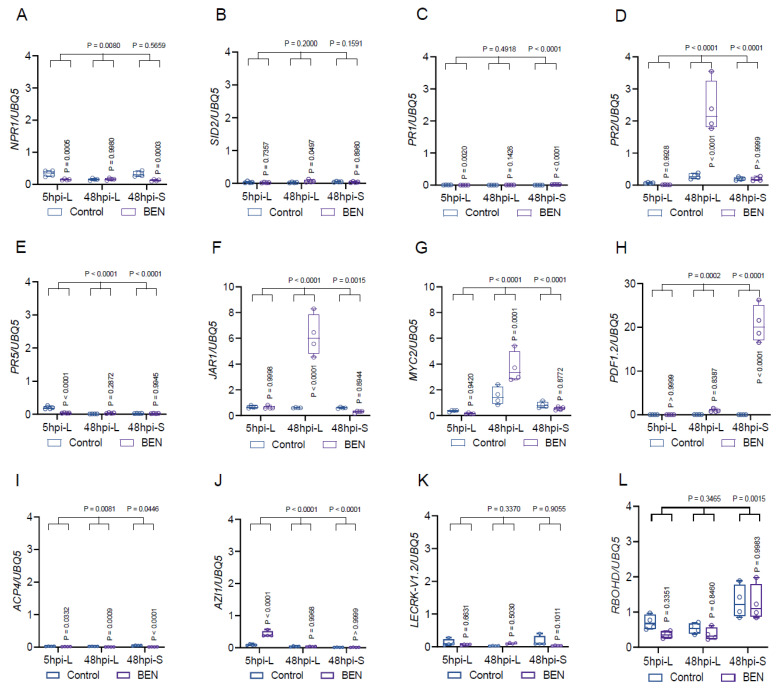
Exogenous BEN-induced plant defense/development gene expression in *Arabidopsis*. (**A**) *NPR1/UBQ5*. (**B**) *SID2/UBQ5*. (**C**) *PR1/UBQ5*. (**D**) *PR2/UBQ5*. (**E**) *PR5/UBQ5*. (**F**) *JAR1/UBQ5*. (**G**) *MYC2/UBQ5*. (**H**) *PDF1.2/UBQ5*. (**I**) *ACP4/UBQ5*. (**J**) *AZI1/UBQ5*. (**K**) *LECRK-V1.2/UBQ5*. (**L**) *RBOHD/UBQ5*. 5hpi-L, locally BEN-infiltrated leaves were collected for total RNA extraction at 5 h post-infiltration. 48hpi-L, locally BEN-infiltrated leaves were collected for total RNA extraction at 48 h post-infiltration. 48hpi-S, systemic non-infiltrated leaves were collected for total RNA extraction at 48 h post-infiltration of BEN to local leaves.

In our preceding experiments, we explored the involvement of the SA pathway in the BEN-mediated plant defense against the fungal pathogen *B. cinerea*, utilizing a pathogen inoculation assay in both wild-type (WT) plants and SA-defective mutants. Our findings revealed that BEN bolstered both local (Figure 7A) and systemic (Figure 8A) resistance in WT plants, while its effects were nullified in *npr1*, *sid2*, and *NahG* OE mutants, which are defective in the SA pathway. These outcomes strongly suggest the indispensable role of the SA pathway in BEN-triggered plant defense. To probe this proposition further, we evaluated the expression of *NPR1* (Figure 9A) and *SID2* (Figure 9B), pivotal genes in the SA pathway, in BEN-treated plants through qRT-PCR at various time points (5 hpi-local, 48 hpi-local, and 48 hpi-systemic). Intriguingly, our data indicated that BEN-induced plant defense may operate independently of *NPR1* (Figure 9A) or *SID2* (Figure 9B) expression. The absence of BEN-induced resistance in SA-defective mutants (Figure 7A) raised an important query: How can we account for this discrepancy? We addressed this query by evaluating the expression of other vital SA-responsive genes—*PR1* (Figure 9C), *PR2* (Figure 9D), and *PR5* (Figure 9E)—in BEN-treated plants using qRT-PCR. Our findings revealed that only *PR2* (Figure 9D) expression may be pivotal for BEN-induced local resistance, but *PR1* (Figure 9C) or *PR5* (Figure 9E) expression is not. This implies that the SA pathway might still contribute to BEN-mediated plant defense, but contingent on specific genes like *PR2* (Figure 9D).

Moreover, our previous demonstrations indicated that WT *Arabidopsis* plants exhibited enhanced local and systemic defense against *B. cinerea* following BEN treatment. Notably, this effect was nullified in some JA-related mutant plants—such as *jar1* and *myc2*—both locally (Figure 7B) and systemically (Figure 8B). These outcomes underscore the pivotal role played by the JA pathway in BEN-induced plant defense. To corroborate this hypothesis, we examined the expression of *JAR1* (Figure 9F), *MYC2* (Figure 9G), and *PDF1.2* (Figure 9H)—key plant defense genes in the JA biosynthesis and signaling pathway—in BEN-treated plants through qRT-PCR at distinct time points (5 hpi-local, 48 hpi-local, and 48 hpi-systemic). The findings unveiled that *JAR1* (Figure 9F) and *MYC2* (Figure 9G) expression is critical for BEN-triggered local defense, whereas *PDF1.2* (Figure 9H) expression is vital for BEN-induced systemic defense. Additionally, we noted a substantial increase in *AZI1* (Figure 9J) expression at 5hpi-L, with no discernible changes in *ACP4* (Figure 9I) and *LECRK-V1.2* (Figure 9K) expression. Importantly, BEN alone failed to stimulate the expression of *RBOHD* (Figure 9L), the ROS-responsive gene, signifying that BEN alone is unlikely to incite the ROS burst seen with flg22, chitin, or BEE (Figure 4I,J).

## 3. Discussion

In this comprehensive study, we conducted a thorough investigation into the multifaceted roles of GD4a, focusing on its capacity to enhance plant growth and combat plant diseases, with particular emphasis on its interactions with the gray mold fungus, *B. cinerea*. Gray mold disease causes substantial yield losses for more than 200 plant species, such as grapes, strawberries, and tomatoes [75,76,77,78]. Utilizing beneficial bacteria as natural resources to assist crop plants in combatting this critical disease holds great promise [79,80,81,82,83,84]. Achieving a comprehensive understanding of the interactions among beneficial bacteria, *B. cinerea* (the causative agent of gray mold), and the host plants is essential for the effective management of this destructive disease. Our research has yielded substantial evidence demonstrating that GD4a plays a pivotal role in promoting the development of plant roots, augmenting shoot biomass, and increasing seed yield in *Arabidopsis*. These beneficial effects are likely attributed to the production of important metabolites, such as indole-3-acetic acid (IAA) and ammonium, by GD4a. Moreover, GD4a has exhibited remarkable efficacy in elevating the host plant’s basal and systemic resistance, including ISR and SAR. This immunomodulatory action is mainly presumably mediated by specific metabolites present in the bacterial extracellular exudate (BEE), including compounds like amino acids, alkaloids, flavonoids, and saponins. It remains unclear whether the metabolites in BEE were released or transported via extracellular vesicles or exosomes [85,86,87,88]. This aspect is not only important to plant science but also vital to human neurodegenerative disease therapy [89,90,91,92]. Intriguingly, our fungal infection assays revealed that GLR3.2 and GLR3.6 are essential for BEN-induced localized plant defense (Figure 7). We also found that GLR3.2, GLR3.3, GLR3.6, and LecRK-VI.2 are crucial for the systemic defense mechanisms triggered by BEN (Figure 8). These findings suggest that either the glutamate receptors or the lectin receptors could serve as potential receptors for BEN in *A. thaliana*, shedding new light on the intricate interplay of plant defense pathways. 

We also found that multiple genes involved in lipid and fatty acid signaling are critical for the BEN-mediated local or/and systemic defense, including *ACP4* for both local and systemic resistance, *DIR1* for local resistance, and *AZI1* and *LTP2* for systemic resistance potentially (Figure 7 and Figure 8). Additionally, GD4a has demonstrated the fine-tuned regulation of essential host plant defense genes, including *PR2*, *JAR1*, *MYC2*, and *PDF1.2*, further underscoring its role in bolstering plant immunity. Furthermore, our research has unveiled GD4a’s direct profound inhibitory effect on the growth of *B. cinerea*, a finding that strongly suggests the potential to decrease the pathogenicity of this fungus. GD4a achieves this by specifically targeting the development of fungal conidia and the formation of new hyphae.

GD4a, as a beneficial bacterium, has demonstrated its ability to produce various metabolites and enhance plant growth through drenching treatment (Figure 1). Although its involvement in nitrogen fixation (N2 fixation) remains uncertain, we have observed increased root hair production in *Arabidopsis* plants when treated with GD4a. This phenomenon could potentially benefit other nitrogen-fixing microorganisms within the root microbiome, providing them with more available sites to initiate nodulation in soil conditions. Furthermore, with the genome sequence of GD4a readily available, there is a great potential for further genetic engineering of GD4a, paving the way for the development of innovative products like nanofertilizers. These nanofertilizers could be applied by spraying them onto plant leaves, offering a novel concept known as “artificial diazotrophic microbiome design” and potentially enhancing biological nitrogen fixation within plant leaves [93,94,95,96].

Our findings also open several intriguing research questions. For instance, what are the specific volatile organic compounds (VOCs) produced by GD4a that effectively combat *B. cinerea*? Our study has convincingly shown that GD4a generates unidentified VOCs that significantly limit the growth of *B. cinerea* in vitro (Figure 2M,N), with an astonishing inhibition rate of approximately 100% (Figure 2O,P). If we can pinpoint the identity of these functional VOCs, there is a substantial opportunity to enhance the control of gray mold disease, particularly in greenhouse and field conditions as well as storage stage. Although further study needs to be developed to identify specific compounds from VOCs, our research does provide additional evidence supporting the potential application of beneficial bacteria VOCs in safeguarding post-harvest fruits, such as tomatoes (Figure 2Q–S). Interestingly, a recent study by Gong et al. demonstrated that airborne defense, involving compounds like methyl-salicylate, could be a bioinspired strategy to combat plant aphid and virus diseases [97]. This raises an intriguing question: could VOCs from beneficial bacteria trigger airborne plant defense mechanisms? As of now, this area of research remains largely unexplored.

In our untargeted metabolomics analysis, we noticed compound 4.00_165.0789n (C_9_H_11_NO_2_) which stood out in both the negative and positive ion modes. BEN, one of the possible isomers of C_9_H_11_NO_2_, was selected for further analysis (Figure 6A,B). We confirmed its effectiveness in activating plant systemic immunity and investigated the related mechanisms in both SAR and ISR in *Arabidopsis* against the bacterial pathogen *Pst DC3000* and the gray mold fungus *B. cinerea* (Figure 6E–H). However, the possibility of other isomers of C_9_H_11_NO_2_ playing roles alongside BEN cannot be ruled out and needs to be further investigated.

Also, our results showed that the detected compounds from BEE by both negative and positive modes included amino acids, alkaloids, flavonoids, and saponins. Their validation, effects, and potentially functional mechanisms also need further substantial study. In more detail, amino acids, the fundamental building blocks of proteins and peptides, play a crucial role in plant growth and immunity. For example, tryptophan acts as a precursor to the plant hormone IAA, which governs both plant growth and resistance to pathogens [98,99]. Glutamate and glutamine are instrumental in activating the salicylic acid (SA) pathway, which imparts resistance against biotrophic pathogens [100]. Proline and glycine betaine act as osmoprotectants, aiding plants in withstanding drought and salinity stress [101]. On one hand, alkaloids, which are nitrogen-containing compounds, exhibit diverse biological activities, including acting as phytoalexins, growth regulators, and neuroactive agents. They can disrupt fungal sterols critical for fungal membrane integrity and induce plant defenses by altering phytohormones, generating ROS, or influencing gene expression. For instance, caffeine enhances plant resistance by elevating jasmonic acid (JA) and ethylene (ET) levels, while nicotine triggers ROS production and activates SA-dependent defense genes in tobacco plants [102]. Flavonoids are polyphenolic compounds with antioxidant, anti-inflammatory, and immunomodulatory properties. They influence the plant microbiome, crucial for plant health and disease resistance, by serving as signals, attractants, or inhibitors. For instance, they induce nodulation in legumes by attracting rhizobia bacteria and inhibit pathogenic fungi by chelating iron or disrupting fungal enzymes [103]. Additionally, saponins, glycosylated compounds with detergent-like properties, function as anti-feedings, anti-nutrients, and toxins against herbivores and pathogens. They also elicit plant defenses by inducing phytohormone production, ROS generation, and defense gene expression. For example, aescin activates SA-dependent immunity in *Brassica napus* and *Arabidopsis* against fungal and bacterial pathogens. Saponins enhance the activity of other antimicrobial compounds by improving their solubility and permeability [104]. Lastly, there is a great potential to utilize beneficial bacteria like GD4a to produce plant-derived-like chemicals for combating human diseases, given that bacteria are more manageable than plants [105,106,107].

## 4. Materials and Methods

### 4.1. Plant Materials and Growth Conditions

In this study, we employed *Arabidopsis thaliana* Columbia ecotype (Col-0) as the wild-type control. Unless specifically noted, all mutants and transgenic plants utilized in our research were based on the Col-0 background [108,109]. Prior to experimentation, homozygous mutant lines were carefully screened according to the previous report [110]. For *Arabidopsis* cultivation, plants were grown in a walk-in chamber under the controlled conditions of 22 °C temperature, 12 h light cycles, and approximately 60% relative humidity. The light intensity was maintained at around 120 μmol/m^2^/s [46]. Tomato cultivars M82 and Micro-Tom as well as strawberry cultivar Monterey-UC were employed in our research. These plants were cultivated in a greenhouse with conditions set at 22 °C temperature, 16 h light cycles, and roughly 45% relative humidity, following previously established protocols [33,47].

### 4.2. Microbial Materials and Culture Conditions

The virulent *Pseudomonas syringae* pv. tomato DC3000 strain (*Pst* DC3000), harboring rifampicin resistance on the chromosome, was cultivated on King’s B Agar (KBA) supplemented with 50 mg/L of rifampicin [111]. *B. amyloliquefaciens* GD4a and *B. amyloliquefaciens* starin D747 were cultured on Tryptic Soy Agar (TSA) at a temperature of 28 °C [15]. D747 is a naturally occurring, beneficial bacterium commonly found in association with various plant parts. It is widely used as a biofungicide to manage both fungal and bacterial plant diseases and serves as the active ingredient in several commercial pest control products. Recognized by the Environmental Protection Agency, the strain has specific manufacturing and concentration guidelines. Its efficacy extends to various application methods, including chemigation and seed treatment. As a result, D747 stands as a promising alternative to chemical pesticides, aligning well with the goals of sustainable agriculture [112]. We used D747 as a positive control for this study. Phytopathogenic fungi including *Botrytis cinerea*, *Fusarium oxysorum*, *Fusarium graminearum*, and *Magnaporthe oryzae*, as well as oomycetes such as *Phytophthora capsici* and *Pythium irregulare*, were all cultivated on Potato Dextrose Agar (PDA) at a temperature of 25 °C [46,47].

### 4.3. Plant Growth Promotion Assay of Arabidopsis Plants

The plant growth promotion assay was conducted following the procedure outlined in the previous study [47], with slight adaptations. In summary, sterile *Arabidopsis* Col-0 seeds were placed onto a 10 cm petri dish containing 25 mL of agar-solidified 0.5× Murashige and Skoog (MS) medium, following a 3-day vernalization at 4 °C. Small 10 µL drops of a bacterial suspension with an OD600 = 0.1 were applied at a distance of 5 cm from the seeds. The inoculated petri dish was then positioned vertically within a plant growth chamber set at 22 °C with a 12 h light/12 h dark cycle to facilitate seed germination and plant growth. After 14 days, measurements were taken for seedling fresh weight and primary root length. For *Arabidopsis* Col-0 plants cultivated in soil pots, they were subjected to weekly drenching treatment with either water as a control or GD4a/D747 for a total of three applications before assessing additional growth parameters such as the rosette width, leaf chlorophyll content, shoot biomass, silique number, and seed weight.

### 4.4. Bacterial Enzyme Activity and Targeted Metabolite Tests

We assessed the enzymatic activities of GD4a/D747 as follows, in accordance with prior methodologies: Pectinase activity was evaluated as described previously [113]. Amylase activity was determined following the procedures outlined in the previous report [114]. Ribonuclease activity was measured using the method detailed in the previous study [115]. Cellulase activity was assayed as per the protocol described in [116]. Catalase activity was determined using biochemical tests commonly employed for the identification of medical bacteria [117]. ACC deaminase activity was assessed following the procedure described previously [118]. Chitinase activity was determined according to previously established methods [119]. The targeted bacterial metabolites associated with beneficial traits, including ammonia, phosphate, IAA, siderophore, HCN, organic acid, exopolysaccharide, acetoin, and diacetyl, were analyzed based on a previous study [46].

### 4.5. Bacterial Extracellular Exudate (BEE) Collection

To collect bacterial extracellular exudate (BEE), we followed a specific procedure outlined in a previous study [46]. Initially, GD4a/D747 bacterial cultures were grown on TSA plates at 28 °C for a period of two days. Subsequently, bacterial cells from each strain (with four plates each) were harvested by washing them off the TSA plates using sterile water, which was then collected into a 50 mL tube. For control purposes, 10 mL of sterile water was similarly used to wash an empty TSA plate. Optionally, the optical density at 600 nm (OD600) was measured in the vortexed solution, although this method was less accurate, or a more accurate approach involved serial drop plating onto a new TSA plate to quantify the absolute cell numbers of each bacterium. The bacterial solution was then subjected to centrifugation at 4 °C for 30 min at 4300 RPM to effectively separate the cell-free supernatant from the bacterial cells. To ensure the supernatant was free of cells, 100 µL of each supernatant was plated onto a new TSA plate so that no bacteria would grow. Generally, each 10 mL of bacterial solution yielded 8 mL of 1× raw BEE. The 1× raw BEE was further concentrated to 10× raw BEE using a freeze dryer. To obtain refined BEE, the raw BEE underwent a methanol purification process, which lasted overnight at −80 °C, followed by centrifugation to remove any residual unresolved residues, primarily proteins.

### 4.6. Fungal Pathogen Infection Assay

For the inoculation of fungal pathogen *B. cinerea* B05.10, we utilized 4- to 6-week-old plant leaves. These leaves were inoculated with a single 10 μL droplet containing 5 × 10^5^ conidia per mL. This inoculation was completed with or without the addition of raw BEE. Following inoculation, the plants were maintained in a cool environment with high humidity. After a period of two to three days, we assessed the disease symptoms by measuring lesion size, a quantification process facilitated using ImageJ software (Version 1.52t 30 January 2020) [120].

### 4.7. Bacterial Pathogen Infection Assay

For the inoculation of the bacterial pathogen *Pst* DC3000, individual leaves underwent syringe injection with *Pst* DC3000 at OD600 = 0.002 (equivalent to 1 × 10^6^ cfu/mL). Alternatively, leaves were subjected to dipping inoculation or spray inoculation with *Pst* DC3000 at OD600 = 1 (equivalent to 5 × 10^8^ cfu/mL), and this inoculation solution contained 0.02% to 0.05% Silwet L-77. After a span of two to three days, we quantified bacterial virulence by assessing bacterial multiplication within the inoculated plant leaves, utilizing the 10 μL spotting technique [121].

### 4.8. Post-Harvesting Tomato Fruit Protection Assay

Mature tomato fruits (Micro-Tom cultivar) were selected randomly and positioned on 9 cm petri dishes. These fruits were subsequently sprayed with *B. cinerea* conidia at a concentration of 5 × 10^5^ conidia/mL. The petri dish, either with or without GD4a growth, was then inverted to replace the original cover, and the fruits were sealed in place using two layers of surgical tape. This sealing ensured a high-humidity environment conducive to *B. cinerea* infection. Representative images were captured seven days later to document the outcomes.

### 4.9. Untargeted Metabolomics Analysis of BEE

The 10× refined BEE samples were combined with a 20 μL solution and mixed into a solution consisting of 80 μL of 5% methanol with 0.1% Formic acid. Subsequently, these samples underwent 20 min of sonication followed by a 30 min centrifugation at 20,000 RPM before being prepared for injection into LC vials. Untargeted analysis was conducted using a Thermo Orbitrap Q-Exactive Plus Orbitrap Mass spectrometer (Thermo Scientific, Waltham, MA, USA), coupled with HPLC separation employing a Poroshell 120 SB-C18 column (2 × 100 mm, 2.7 µm particle size) and a WPS 3000 LC system. The solvent gradient included solvent A, composed of H_2_O with 0.1% Formic acid, and solvent B, consisting of MeOH with 0.1% Formic Acid, at a flow rate of 200 µL/min. The gradient began with 5% solvent B, underwent a linear increase to 95% B at 15 min, held at 95% B for 1 min, returned to 5% B at 17 min, and finally equilibrated at 5% B until the 30 min mark. Each sample was injected in a volume of 5 µL, and the top 5 ions were chosen for data-dependent analysis with a 15 s exclusion window [122]. In the untargeted results analysis, which involved feature selection, database comparison, and statistical processing, samples were assessed in Progenesis QI (https://www.nonlinear.com/progenesis/qi/, accessed on 20 October 2022) with the pooled sample runs serving as the basis for feature alignment.

### 4.10. Analysis of Local Basal Defense Induced by Benzocaine (BEN) in Arabidopsis Plants against Phytopathogen Infection

Benzocaine (BEN) was selected based on the above untargeted metabolite analysis. Local leaves were pre-infiltrated with either sterile water or a 0.1 mM BEN solution five hours before inoculation with the bacterial pathogen *Pst* DC3000 (infiltration at 10^6^ CFU/mL) or the fungal pathogen *B. cinerea* B05.10 (10^5^ conidia/mL). The sampling of local leaves for counting the *Pst DC3000* number or measuring the lesion size was conducted 2–3 days post-infection, following the method described in [123].

### 4.11. Induction of Systemic Resistance (ISR) of Arabidopsis Plants

We conducted the induction of ISR in *Arabidopsis* plants following the method outlined in a previous study [124], with some adjustments. To be specific, for the GD4a or D747 treatments, we drenched each pot with 10 mL of the respective cell suspension at a concentration of 5 × 10^8^ CFU/mL around the roots of *Arabidopsis* plants. One week later, plants with the respective treatments were subjected to the challenge-inoculation with the bacterial phytopathogen *Pst* DC3000 or the fungal phytopathogen *B. cinerea* B05.10.

### 4.12. Induction of Systemic Resistance (ISR) of Strawberry Plants

We applied the ISR induction method to strawberry plants with some adjustments, following the procedure outlined previously [124]. To elaborate, for the GD4a or D747 treatments, we drenched each pot with 20 mL of the respective cell suspension at a concentration of 5 × 10^8^ CFU/mL, around the strawberry roots. After one week, plants subjected to these treatments were challenged with either the bacterial phytopathogen *Pst* DC3000 or the fungal phytopathogen *B. cinerea* B05.10. For *Pst* DC3000 infection, above-ground plant leaves underwent syringe injection with *Pst* DC3000 at OD600 = 0.002 (equivalent to 1 × 10^6^ cfu/mL). After a span of two to three days, we quantified bacterial virulence by assessing bacterial multiplication within the inoculated plant leaves, utilizing the 10 μL spotting technique [121]. For *B. cinerea* B05.10 infection, above-ground plant leaves underwent fungal disc inoculation following a previous study [33].

### 4.13. Analysis of SAR of Arabidopsis Plants against Bacterial or Fungal Pathogens

The local leaves were subjected to infiltration with either sterile water, GD4a/D747 (at 10^7^ CFU/mL), 1× raw BEE, or 1 mM BEN two days prior to injecting the systemic leaves with the virulent *Pst* DC3000 bacteria (at 10^5^ CFU/mL) or the virulent fungi *B. cinerea* B05.10 (10^5^ conidia/mL). The systemic leaves were then sampled for the analysis within 2–3 days post-infection, following the established protocol [123].

### 4.14. PAMP-Triggered Immunity (PTI) Related Assays

For the ROS burst assay, we followed the procedure as detailed in [46,125]. In brief, *Arabidopsis* leaf discs were first harvested and incubated overnight in sterile water for equilibration. The leaf discs were then subjected to a specific stimulus such as BEE. Reactive Oxygen Species (ROS) produced in response were detected using a chemiluminescence method employing luminol. Data were collected through a plate reader and analysis focused on comparing treated and untreated samples to quantify ROS levels.

The detection of superoxide anion (•O^2−^), a group of ROS, using nitroblue tetrazolium (NBT) staining followed a modified version of the procedure outlined in a previous study [126]. To summarize, plant leaves were harvested, meticulously cleaned, and then exposed to NBT solution (0.1% *w/v* NBT, 10 mM sodium azide NaN_3_, 50 mM potassium phosphate K3PO4, pH 6.4, 0.05% *v/v* silwet 77) in the presence of light at room temperature. After 30 min, the stained leaves underwent a series of ethanol washes to halt the reaction and eliminate chlorophyll. Superoxide anions manifested as a blue stain, which was subsequently photographed. For destaining, leaves could be treated at 40 °C with 95% ethanol.

Hydrogen peroxide (H_2_O_2_), a group of ROS, detection by diaminobenzidine tetrahydrochloride (DAB) staining was carried out with some modifications based on the previous study [127]. Briefly, plant leaves were gathered, thoroughly cleaned, and then exposed to a fresh DAB solution (1 mg/mL diaminobenzidine, pH adjusted to 3.0 with 0.2 M HCl to dissolve DAB, and 0.05% *v/v* silwet 77) in darkness at the room temperature. After 4 h, the stained leaves were cleared using 95% ethanol and preserved in 50% ethanol. Hydrogen peroxide was visualized as a dark-brown precipitate and photographed. For destaining, leaves could be treated at 40 °C with 95% ethanol.

A callose deposition examination was conducted with modifications following the procedure outlined in a previous study [127]. Briefly, plant leaves were collected, thoroughly cleaned, and subjected to vacuum infiltration in a destaining solution (acetic acid/ethanol = 5:95, *v*/*v*). After 10 min, leaf chlorophyll was removed in a water bath at 60 °C. Thirty minutes later, chlorophyll-free leaves were rinsed and soaked in an aniline blue staining solution (0.01% aniline blue, *w*/*v*, in 150 mM K_2_HPO_4_) in the dark. After 2 h, the leaves were rinsed in water and examined under an epifluorescence microscope with a UV excitation filter. The callose deposition was quantified using Image J software [120].

### 4.15. qRT-PCR Assay

*Arabidopsis* Col-0 leaves underwent infiltration with either water or 1 mM BEN. After 5 h or 48 h, locally infiltrated leaves (referred to as 5hpi-L and 48hpi-L) or systemic, non-infiltrated leaves (48hpi-L) were harvested to extract total RNA. These total RNA samples were subjected to DNase I treatment to eliminate any potential DNA remnants. The resulting DNA-free RNA samples were employed for real-time PCR analysis, utilizing specific primers designed for various *Arabidopsis* genes, with *UBQ5* serving as the internal reference gene.

### 4.16. Statistical Analysis

All statistical analyses were conducted using GraphPad Prism 9 software. Data were appropriately analyzed through two-tailed unpaired *t*-tests and/or post hoc one-way ANOVA Tukey’s multiple comparisons tests and/or two-way ANOVA Šídák’s multiple comparisons tests. The data were presented using box and whisker plots, indicating the range from the minimum to the maximum value and displaying all data points. Exact *p*-values were provided in each corresponding graph. Each experiment was repeated thrice at least with similar trends.

## 5. Conclusions

**Figure 10:**
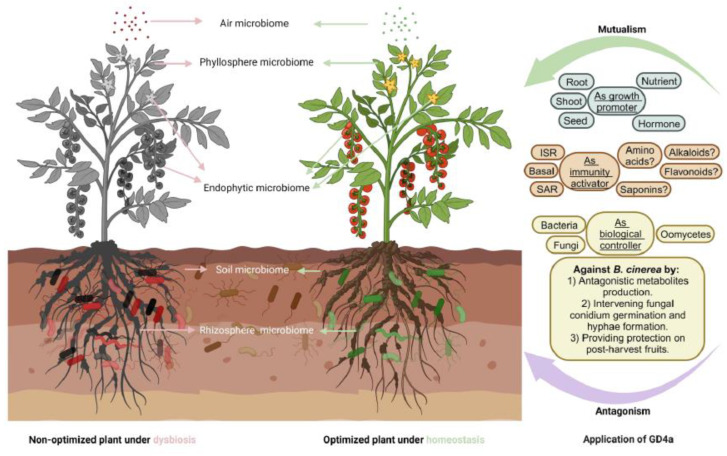
A proposed model outlining how GD4a contributes to safeguarding plant fitness. GD4a can function as a plant growth promoter, a plant immunity activator, and a plant pathogen biological controller. Specifically, GD4a effectively controls the gray mold fungus *B. cinerea* by producing functional metabolites that directly and indirectly (ISR and SAR) inhibit the fungal conidial germination and hyphae formation and thus provide excellent protection for plants during the growth season and the post-harvest stage, such as storage and transportation processes.

In summary, the discovery about beneficial microbes like GD4a has great potential for protecting and preserving plants during the growth stage and after harvesting. Figure 10 illustrates the potential functional mechanisms and practical applications of this research. By understanding how beneficial microbes function, this study expands our knowledge and inspires new disease control strategies. The introduction of the “plant fitness tetrahedron” model, as coined by Yang et al. in our recent work, adds a holistic approach to plant health [53]. This innovative approach centered around beneficial microorganisms like GD4a, promises new avenues for enhancing plant health and productivity [79,80]. Future research on GD4a could focus on unraveling and validating more specific metabolites involved in its interaction with diverse plant species, shedding light on the biochemical pathways that contribute to plant fitness protection. For instance, a critical aspect involves exploring how GD4a-induced changes in plant metabolites and related defense pathways influence the overall health and resilience of crops [128]. The related investigation is essential for fine-tuning the practical applications of GD4a in agriculture.

## Data Availability

All the raw data of this study are available from the corresponding author upon a reasonable request.
